# The importance of harmonising diagnostic criteria sets for pathological grief

**DOI:** 10.1192/bjp.2019.240

**Published:** 2021-09

**Authors:** Lonneke I. M. Lenferink, Paul A. Boelen, Geert E. Smid, Muirne C. S. Paap

**Affiliations:** 1 Postdoctoral Researcher, Department of Clinical Psychology and Experimental Psychopathology, Faculty of Behavioral and Social Sciences, University of Groningen; and Postdoctoral Researcher, Department of Clinical Psychology, Faculty of Social Sciences, Utrecht University, The Netherlands; 2 Professor, Department of Clinical Psychology, Faculty of Social Sciences, Utrecht University; Professor, ARQ National Psychotrauma Centre; and Clinical Psychologist, Foundation Centrum ‘45, The Netherlands; 3 Psychiatrist, ARQ National Psychotrauma Centre; Psychiatrist, Foundation Centrum ’45; and Professor, University of Humanistic Studies, The Netherlands; 4 Assistant Professor, Department of Inclusive and Special Needs Education, Faculty of Behavioural and Social Sciences, University of Groningen; Assistant Professor, Department of Child and Family Welfare, Faculty of Behavioural and Social Sciences, University of Groningen, The Netherlands; and Researcher, Department of Research and Development, Clinic Mental Health and Addiction, Oslo University Hospital, Norway

**Keywords:** Persistent complex bereavement disorder, prolonged grief disorder, ICD-11, DSM-5, complicated grief

## Abstract

Five diagnostic criteria sets for pathological grief are currently used in research. Studies evaluating their performance indicate that it is not justified to generalise findings regarding prevalence rates and predictive validity across studies using different diagnostic criteria of pathological grief. We provide recommendations to move the bereavement field forward.

Yearning for a significant other who has died, being preoccupied with the loss and circumstances surrounding it, and sadness are reactions frequently experienced by bereaved people. Most people adapt to the death of a significant other over time.^1^ When grief reactions interfere with daily life tasks for a prolonged period of time following the death, a diagnosis of a grief disorder (i.e. pathological grief) might apply. Factor analytic studies and latent class analyses have shown that pathological grief reactions are related to, yet distinguishable from, symptoms of depression and post-traumatic stress disorder.^2,3^ In addition, it has been shown that people with pathological grief benefit from grief-focused treatment more than non-grief-focused treatment.^4,5^ A meta-analysis has shown that one out of ten bereaved people are at risk for experiencing pathological grief after a natural death (e.g. owing to illness).^6^ Caution is, however, warranted when interpreting the findings of this meta-analysis, because of several limitations. The included studies varied in terms of study sample (e.g. representative versus non-representative samples), operationalisation of pathological grief (i.e. different diagnostic criteria sets for pathological grief were used) and measurement of pathological grief (i.e. different surveys and few clinical diagnostic interviews were used).

Efforts from clinical and research experts have led to the inclusion of grief disorders in recent editions of the two most frequently used diagnostic classification systems in mental healthcare: the DSM-5^7^ and the ICD-11.^8^ Earlier, Prigerson *et al*^9^ proposed a set of criteria for prolonged grief disorder (PGD; hereafter referred to as PGD-2009) and Shear *et al*^10^ proposed a different set for complicated grief. Psychometric properties of the latter two criteria sets have been evaluated with methods from classical test theory and item response theory.^9,11^ Independently, both research groups concluded that the criteria sets they proposed for pathological grief adequately differentiate bereaved people with non-pathological grief from those with pathological grief.

The ten criteria for PGD-2009 and 12 criteria for complicated grief were eventually not included in the DSM-5 and ICD-11. Instead, a combination of these two sets, named persistent complex bereavement disorder (PCBD), was included as one of the ‘other specified trauma- and stressor-related disorders’ and as a condition for further study in Section III of the DSM-5.^7^ Because of the preliminary nature of criteria sets in Section III, it can be expected that the operationalisation and/or naming of PCBD will change in future revisions of the DSM. PCBD can be diagnosed when, following the death of a significant other, at least one of four separation distress symptoms and at least six of 12 symptoms of reactive distress and social/identity disruption are present to the point of impairment at least 12 months (6 months for children) after the death.^7^ In addition, PGD was recently included in the ICD-11.^8^ PGD can be diagnosed 6 months post-loss, when at least one out of two separation distress symptoms combined with at least one out of ten accompanying symptoms are present to the point of impairment.^8,12,13^

PCBD as per DSM-5 seems to be a compromise between the two proposed diagnostic criteria sets by Prigerson *et al*^9^ and Shear *et al*,^10^ augmented with three additional criteria.^14^ In a beta-draft of the ICD-11,^15^ a version of PGD was introduced encompassing seven criteria (hereafter referred to as beta-draft ICD-11 PGD). The final version of PGD as per ICD-11^8^ (hereafter referred to as ICD-11 PGD) encompasses 12 diagnostic criteria. Beta-draft ICD-11 PGD and ICD-11 PGD seem to be based on Prigerson *et al*’s^9^ PGD proposal, but with some alterations.^12^ Thus, over the past decade, five different criteria sets have been proposed in the literature. Figure 1 provides an overview of the similarities and differences between these five diagnostic criteria sets (see also Table 1 in Supplementary material available at https://doi.org/10.1192/bjp.2019.240).

**Fig. 1:**
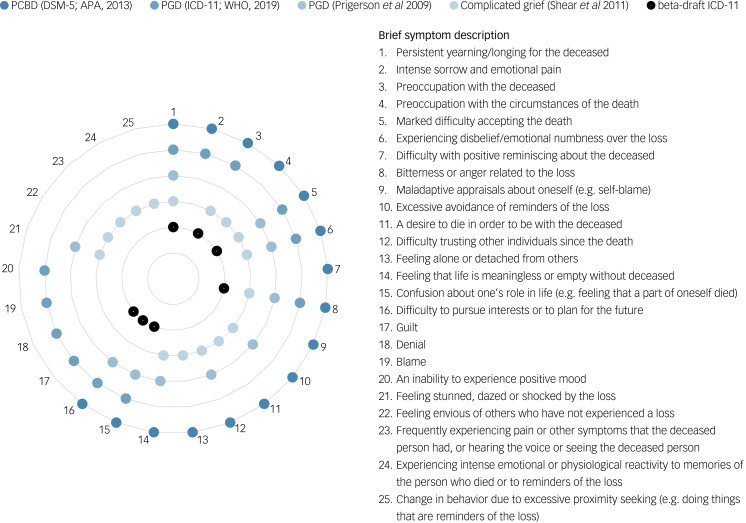
Similarities and differences between five diagnostic criteria sets of pathological grief. For illustrative purposes, the following compound complicated grief criteria are displayed as two symptoms rather than one symptom: criterion B2, ‘Frequent intense feeling of loneliness or like life is empty or meaningless without the person who died’, displayed as symptoms 13 and 14; criterion C2, ‘Recurrent feeling of disbelief or inability to accept the death, like the person cannot believe or accept that their loved one is really gone’, displayed as symptoms 5 and 6; criterion C3, ‘Persistent feeling of being shocked, stunned, dazed, or emotionally numb since the death’, displayed as symptoms 6 and 21; criterion C5, ‘Persistent difficulty trusting or caring about other people or feeling intensely envious of others who have not experienced a similar loss’, displayed as symptoms 12 and 22; and criterion C8, ‘Change in behaviour owing to excessive avoidance or the opposite, excessive proximity-seeking, e.g. refraining from going places, doing things or having contact with things that are reminders of the loss, or feeling drawn to reminders of the person, such as wanting to see, touch, hear or smell things to feel close to the person who died’, displayed as symptoms 10 and 25. PCBD, persistent complex bereavement disorder; PGD, prolonged grief disorder.

Research has shown that different prevalence rates for pathological grief are found when applying different criteria sets. In addition, evidence in support of predictive validity for some criteria sets (i.e. complicated grief and ICD-11 PGD) is lacking. Maciejewski *et al*^16^ showed that interview-based DSM-5 PCBD, PGD-2009 and beta-draft ICD-11 PGD in a community bereaved sample are similar in terms of prevalence rates (~10%) and predictive validity (i.e. presence of diagnosis significantly predicted more functional impairment and lower quality of life over time), whereas complicated grief showed higher prevalence rates (30%) and lacked predictive validity (i.e. presence of a complicated grief diagnosis did not predict functional impairment or decreased quality of life over time).

Cozza *et al*^17^ and Mauro *et al*^18^ examined whether different diagnostic criteria sets of pathological grief (survey-based or interview-based) resulted in differences regarding detecting clinical cases in a sample of bereaved families of military personnel and in a treatment-seeking sample, respectively. A predefined criterion for ‘caseness’ was used; scores of 30 or higher on the Inventory of Complicated Grief were considered an indication of ‘caseness’ and scores below 20 indicated ‘non-caseness’. People whose Inventory of Complicated Grief score fell between 20 and 30 were not included in the studies. Both Cozza *et al*^17^ and Mauro *et al*^18^ concluded that complicated grief criteria were superior when it comes to correctly identifying clinical cases (i.e. over 90% of clinical cases were detected), whereas DSM-5 PCBD and PGD-2009 criteria were too stringent (i.e. 50–70% of clinical cases were detected). Mauro *et al*^13^ compared the diagnostic criteria of interview-based PGD-2009 with ICD-11 PGD in a treatment-seeking bereaved sample, using similar methods as Cozza *et al*^17^ and Mauro *et al*,^18^ and concluded that ICD-11 PGD outperformed PGD-2009 (identifying 96 *v*. 59% clinical cases). Importantly, the marker for ‘caseness’ used by Cozza *et al*^17^ and Mauro *et al*^13,18^ sparked a debate in which scholars expressed serious methodological concerns about excluding people with scores between 20 and 30 on the Inventory of Complicated Grief from the analyses, and argued that distinguishing normal from pathological grief for these ‘borderline cases’ is the real challenge.^19,20^ In response, Cozza *et al*^21^ reanalysed their data including the borderline cases and concluded that ICD-11 PGD and complicated grief criteria outperformed DSM-5 PCBD and PGD-2009 criteria in terms of identifying ‘clinical caseness’.

Two studies have shown that applying diagnostic criteria for DSM-5 PCBD versus ICD-11 PGD results in substantially different findings in terms of prevalence and predictive validity. More specifically, prevalence rates were shown to be at least two times higher using the ICD-11 PGD criteria compared with DSM-5 PCBD criteria.^22,23^ However, increasing the number of symptoms needed to meet ICD-PGD criteria to at least five accompanying symptoms improved agreement in prevalence rates between DSM-5 and ICD-11 pathological grief.^23^ Furthermore, people meeting (versus not meeting) self-rated criteria for DSM-5 PCBD at baseline reported significantly higher pathological grief, depression and post-traumatic stress symptom levels 1 year later when controlling for baseline symptom levels, whereas ‘caseness’ of self-rated criteria for ICD-11 PGD at baseline did not predict the intensity of these symptoms 1 year later.^22^

It should be noted that most studies evaluating the psychometric properties of the diagnostic criteria sets for pathological grief used (a selection of) items that were similar to the diagnostic criteria that they intended to assess, but these items had not all been developed to assess these criteria. For instance, Mauro *et al*^13^ used one item (i.e. ‘trouble accepting’) of the Structured Clinical Interview for Complicated Grief to assess two ICD-11 PGD criteria (i.e. ‘denial’ and ‘difficulty accepting the death’) and Boelen *et al*^22,23^ used items from a depression measure to assess some ICD-11 PGD and DSM-5 PCBD criteria. Moreover, certain measures that were developed to assess a specific criteria set of pathological grief (e.g., the Structured Clinical Interview for Complicated Grief) are not well validated; for instance, psychometric properties were not evaluated across samples that differ with respect to cultural background, age and mode of death. In addition, current measures used to assess pathological grief criteria differ in response scales (frequency versus severity) and delivery format (survey versus interview), which limits comparability of findings across studies. Lastly, comparability between prevalence rates and predictive validity across studies is also hindered by the lack of a gold standard for defining ‘caseness’ of pathological grief, which in turn leads to differences in findings.

To overcome the limitations of prior comparative studies and to move the bereavement field forward, we propose the following two objectives. First, it is pivotal that researchers explicitly and consistently report which pathological grief criteria they have used in their study to avoid confusion or misinterpretation. As noted, research has indicated that different diagnostic criteria sets yield different prevalence rates and vary in terms of predictive validity. It is therefore not justified to generalise findings regarding prevalence rates and predictive validity across studies using different diagnostic criteria of pathological grief, and researchers should acknowledge this when interpreting their findings.

Second, it is essential for researchers to use instruments that are intended to assess diagnostic criteria of pathological grief when drawing conclusions about diagnostic performance. Empirical evidence regarding performance of diagnostic criteria sets of pathological grief is primarily based on self-report questionnaires, which may overestimate symptom levels (as shown in depression research^24^). Using clinical diagnostic interviews that tap into both DSM-5 PCBD and ICD-11 PGD diagnostic criteria, but ideally include all criteria sets, measured with uniform response scales, would allow researchers to overcome limitations of prior comparative studies and would allow a direct comparison of the diagnostic performance of the different diagnostic criteria sets for pathological grief. Furthermore, the performance of diagnostic criteria sets should be evaluated across different samples of bereaved people, varying in terms of, for example, mode of death, age, recruitment source (treatment-seeking versus non-treatment-seeking people), time frame since death and cultural background.

In summary, it is advised that researchers use clinical diagnostic interviews to further evaluate the validity and utility of pathological grief criteria. This could inform future updates of the psychiatric classification systems in which diagnostic criteria sets for pathological grief are harmonised. This is urgently needed in order to reach consensus on criteria that correctly identify bereaved people in need of professional support and, consequently, to prevent unnecessary pathologisation of grief reactions.

## Supplementary material

For supplementary material accompanying this paper visit https://doi.org/10.1192/bjp.2019.240. click here to view supplementary material

## References

[1] Nielsen M, Carlsen A, Neergaard M, Bidstrup P, Guldin M. Looking beyond the mean in grief trajectories: a prospective, population-based cohort study. Soc Sci Med 2019; 232: 460–9. doi:10.1016/j.socscimed.2018.10.007 31230666

[2] Boelen PA, van de Schoot R, van den Hout MA, de Keijser J, van den Bout J. Prolonged grief disorder, depression, and posttraumatic stress disorder are distinguishable syndromes. J Affective Disord 2010; 125(1–3): 374–8. doi:10.1016/j.jad.2010.01.076 20189657

[3] Lenferink LIM, de Keijser J, Smid GE, Djelantik AAAMJ, Boelen PA. Prolonged grief, depression, and posttraumatic stress in disaster-bereaved individuals: latent class analysis. Eur J Psychotraumatol 2017; 8(1): 1298311. doi:10.1080/20008198.2017.12983114 28451067 PMC5399993

[4] Boelen PA, de Keijser J, van den Hout MA, van den Bout J. Treatment of complicated grief: a comparison between cognitive-behavioral therapy and supportive counseling. J Consulting Clin Psychol 2007; 75(2): 277–4. doi:10.1037/0022-006X.75.2.277 17469885

[5] Shear K, Frank E, Houck PR, Reynolds CF 3rd. Treatment of complicated grief: a randomized controlled trial. JAMA 2005; 293(21): 2601–8. doi:10.1001/jama.293.21.2601 15928281 PMC5953417

[6] Lundorff M, Holmgren H, Zachariae R, Farver-Vestergaard I, O'Connor M. Prevalence of prolonged grief disorder in adult bereavement: a systematic review and meta-analysis. J Affect Disord 2017; 12: 138–49. doi:10.1016/j.jad.2017.01.030 28167398

[7] American Psychiatric Association (APA). Diagnostic and Statistical Manual of Mental Disorders (5th ed.). American Psychiatric Publishing, 2013.

[8] World Health Organization (WHO). ICD-11 Prolonged Grief Disorder Criteria. WHO, 2019 (https://icd.who.int/browse11/l-m/en#/http://id.who.int/icd/entity/1183832314).

[9] Prigerson HG, Horowitz MJ, Jacobs SC, Parkes CM, Aslan M, Goodkin K, Prolonged grief disorder: psychometric validation of criteria proposed for DSM-V and ICD-11. PLoS Med 2009; 6(8): e1000121. doi:10.1371/journal.pmed.1000121 19652695 PMC2711304

[10] Shear MK, Simon N, Wall M, Zisook S, Neimeyer R, Duan N, Complicated grief and related bereavement issues for DSM-5. Depress Anxiety 2011; 28(2): 103–17. doi:10.1002/da.20780 21284063 PMC3075805

[11] Simon NM, Wall MM, Keshaviah A, Dryman M, LeBlanc N, Shear MK. Informing the symptom profile of complicated grief. Depress Anxiety 2011; 28(2): 118–26. doi:10.1002/da.20775 21284064 PMC3079952

[12] Killikelly C, Maercker A. Prolonged grief disorder for ICD-11: the primacy of clinical utility and international applicability. Eur J Psychotraumatol 2017; 8(Suppl 6): 1476441. doi:10.1080/20008198.2018.1476441 29887976 PMC5990943

[13] Mauro C, Reynolds C, Maercker A, Skritskaya N, Simon N, Zisook S, Prolonged grief disorder: clinical utility of ICD-11 diagnostic guidelines. Psychol Med 2019; 49(5): 861–7. doi:10.1017/S0033291718001563 29909789

[14] Boelen PA, Prigerson HG. Commentary on the inclusion of persistent complex bereavement-related disorder in DSM-5. Death Stud 2012; 36(9): 771–94. doi:10.1080/07481187.2012.706982 24563927

[15] Maercker A, Brewin CR, Bryant RA, Cloitre M, van Ommeren M, Jones LM, Diagnosis and classification of disorders specifically associated with stress: proposals for ICD-11. World Psychiatry 2013; 12(3): 198–206. doi:10.1002/wps.20057 24096776 PMC3799241

[16] Maciejewski PK, Maercker A, Boelen PA, Prigerson HG. ‘Prolonged grief disorder’ and ‘persistent complex bereavement disorder’, but not ‘complicated grief’, are one and the same diagnostic entity: an analysis of data from the Yale Bereavement Study. World Psychiatry 2016; 15: 266–75. doi:10.1002/wps.20348 27717273 PMC5032512

[17] Cozza SJ, Fisher JE, Mauro C, Zhou J, Ortiz CD, Skritskaya N, Performance of DSM-5 persistent complex bereavement disorder criteria in a community sample of bereaved military family members. Am J Psychiatry 2016; 173: 919–29. doi:10.1176/appi.ajp.2016.15111442 27216262

[18] Mauro C, Shear M, Reynolds C, Simon N, Zisook S, Skritskaya N, Performance characteristics and clinical utility of diagnostic criteria proposals in bereaved treatment-seeking patients. Psychol Med 2017; 47(4): 608–15. doi:10.1017/S0033291716002749 27821201

[19] Maciejewski PK, Prigerson HG. Prolonged, but not complicated, grief is a mental disorder. Br J Psychiatry 2017; 211: 189–91. doi:10.1192/bjp.bp.116.196238 28970298

[20] Smid GE, Boelen PA. Performance of complicated grief criteria. Am J Psychiatry 2016; 173: 1149. doi:10.1176/appi.ajp.2016.16060704 27798990

[21] Cozza S, Shear M, Reynolds C, Fisher J, Zhou J, Maercker A, Optimizing the clinical utility of four proposed criteria for a persistent and impairing grief disorder by emphasizing core, rather than associated symptoms. Psychol Med 2019: 1–8. doi:10.1017/S0033291719000254 PMC7025160 30829195

[22] Boelen PA, Lenferink LIM, Nickerson A, Smid GE. Evaluation of the factor structure, prevalence, and validity of disturbed grief in DSM-5 and ICD-11. J Affective Disord 2018; 240: 79–97. doi:10.1016/j.jad.2018.07.041 30059938

[23] Boelen PA, Lenferink LIM, Smid GE. Further evaluation of the factor structure, prevalence, and concurrent validity of DSM-5 criteria for persistent complex bereavement disorder and ICD-11 criteria for prolonged grief disorder. Psychiatry Res 2019; 3: 206–10. doi: 10.1016/j.psychres.2019.01.006 30654306

[24] Lim G, Tam W, Lu Y, Ho C, Zhang M, Ho R. Prevalence of depression in the community from 30 countries between 1994 and 2014. Sci Rep 2018; 8(1): 2861. doi:10.1038/s41598-018-21243-x 29434331 PMC5809481

