# Supplementary material for: The importance of harmonising diagnostic criteria sets for pathological grief
Source: Br J Psychiatry. 2021 Sep;219(3):473–6. doi: 10.1192/bjp.2019.240 (PMC8387857; doi:10.1192/bjp.2019.240)
Supplement: Supplementary file 1 [file S000712501900240Xsup001.docx]

**Supplementary Material**

**Table 1.** Similarities and differences between five diagnostic criteria sets of pathological grief

| Symptoms | DSM-5 Persistent complex bereavement disorder (7) | ICD-11 Prolonged grief disorder (8) | Prolonged grief disorder – 2009 (9) | Complicated grief (10) | Beta-draft ICD-11 Prolonged grief disorder (15) |
| --- | --- | --- | --- | --- | --- |
| Persistent yearning/longing for the deceased. | X | X | X | X | X |
| Intense sorrow and emotional pain | X | X |  |  |  |
| Preoccupation with the deceased | X | X |  | X | X |
| Preoccupation with the circumstances of the death | X |  |  | X |  |
| Marked difficulty accepting the death | X | X | X | X | X |
| Experiencing disbelief/emotional numbness over the loss | X | X | X | X |  |
| Difficulty with positive reminiscing about the deceased. | X |  |  |  |  |
| Bitterness or anger related to the loss | X | X | X | X | X |
| Maladaptive appraisals about oneself (e.g., self-blame) | X |  |  |  |  |
| Excessive avoidance of reminders of the loss | X |  | X | X |  |
| A desire to die in order to be with the deceased | X |  |  | X |  |
| Difficulty trusting other individuals since the death | X |  | X | X |  |
| Feeling alone or detached from others | X |  |  | X |  |
| Feeling that life is meaningless or empty without the deceased | X |  | X | X |  |
| Confusion about one’s role in life (e.g., feeling that a part of oneself died) | X | X | X |  | X |
| Difficulty to pursue interests or to plan for the future | X | X | X |  | X |
| Guilt |  | X |  |  | X |
| Denial |  | X |  |  |  |
| Blame |  | X |  |  |  |
| An inability to experience positive mood |  | X |  |  |  |
| Feeling stunned, dazed or shocked by the loss |  |  | X | X |  |
| Feeling envious of others who have not experienced a loss |  |  |  | X |  |
| Frequently experiencing pain or other symptoms that the deceased person had, or hearing the voice or seeing the deceased person |  |  |  | X |  |
| Experiencing intense emotional or physiological reactivity to memories of the person who died or to reminders of the loss |  |  |  | X |  |
| Change in behavior due to excessive proximity seeking (e.g., doing things that are reminders of the loss) |  |  |  | X |  |
